# Open-Label Study of Duloxetine for the Treatment of Obsessive-Compulsive Disorder

**DOI:** 10.1093/ijnp/pyu062

**Published:** 2015-01-29

**Authors:** Darin D. Dougherty, Andrew K. Corse, Tina Chou, Amanda Duffy, Amanda R. Arulpragasam, Thilo Deckersbach, Michael A. Jenike, Nancy J. Keuthen

**Affiliations:** Division of Neurotherapeutics, Department of Psychiatry, Massachusetts General Hospital, Harvard Medical School, Charlestown, MA (Drs Dougherty, Corse, Chou, Duffy, Arulpragasam, and Deckersbach); OCD and Related Disorders Program, Massachusetts General Hospital, Boston, MA (Drs Dougherty, Jenike, and Keuthen); Department of Psychology, Harvard University, Cambridge, MA (Dr Chou); Department of Neuroscience, Brown University, Providence, RI (Dr Duffy).

**Keywords:** duloxetine, obsessive-compulsive disorder, serotonin-norepinephrine reuptake inhibitors, compulsions, obsessions

## Abstract

**Background:**

This study sought to investigate the efficacy of duloxetine for the treatment of obsessive-compulsive disorder (DSM-IV).

**Methods:**

Twenty individuals were enrolled in a 17-week, open-label trial of duloxetine at Massachusetts General Hospital. Data were collected between March 2007 and September 2012. Study measures assessing obsessive-compulsive disorder symptoms, quality of life, depression, and anxiety were administered at baseline and weeks 1, 5, 9, 13, and 17. The primary outcome measures were the Yale-Brown Obsessive Compulsive Scale and Clinical Global Improvement scale.

**Results:**

For the 12 study completers, pre- and posttreatment analyses revealed significant improvements (*P*<.05) on clinician- and self-rated measures of obsessive-compulsive disorder symptoms and quality of life. Among the 12 completers, more than one-half (n=7) satisfied full medication response criteria. Intention-to-treat analyses (n=20) showed similar improvements (*P*<.05) on primary and secondary study outcome measures.

**Conclusion:**

The results of this study suggest that duloxetine may provide a significant reduction in symptoms for patients with obsessive-compulsive disorder.

ClinicalTrials.gov NCT00464698; http://clinicaltrials.gov/ct2/show/NCT00464698?term=NCT00464698&rank=1.

## Introduction

First-line treatment options for obsessive compulsive disorder (OCD) include serotonin reuptake inhibitors (SRIs) and behavioral therapy. Currently, the SRIs are the primary pharmacotherapy treatment option for OCD (Dougherty et al., 2004). Multiple randomized, placebo-controlled, double-blind studies support the efficacy of SRIs as treatment for OCD (Karabanow et al., 1977; Montgomery, 1980; Perse et al., 1987; Goodman et al., 1989a; Jenike et al., 1990; Goodman et al., 1996; Kronig et al., 1999). Because serotonergic agents have been shown to be the most effective treatment for OCD, serotonin-norepinephrine reuptake inhibitors have been studied for the treatment of OCD as well. A small body of evidence supports the efficacy of venlafaxine for the treatment of OCD. Previous studies include small open-label studies (Rauch et al., 1996) and case reports (Zajecks et al., 1990; Anath et al., 1995; Grossman et al., 1996). One double-blind, head-to-head study compared venlafaxine with paroxetine, finding that the agents were equally efficacious (Denys et al., 2003).

Duloxetine, a new serotonin-norepinephrine reuptake inhibitor (SNRI), has not yet been studied as a treatment option for OCD. It is believed that because duloxetine has strong serotonin reuptake inhibition properties, it will be an effective treatment for OCD. Monotherapeutic pharmacological treatment is currently limited to SRIs, so data supporting the use of other agents could be of tremendous benefit. This study proposes to assess the effects of duloxetine for the treatment of OCD via an open-label clinical trial in 20 patients with OCD.

## Methods

### Participants

Twenty participants with OCD were enrolled in a 17-week, open-label treatment trial with duloxetine. The recruitment occurred under supervision of the Massachusetts General Hospital OCD Clinic and Research Unit. All study protocols and consents were approved by the local institutional review board prior to study initiation and enrollment. Subjects were recruited through clinician referrals as well as through hospital-approved advertisements posted in newspapers, on the internet, and on hospital bulletin boards. This study was also listed in the ClinicalTrials.gov registry. All participants signed informed consent statements prior to study engagement.

Participants met the following study inclusion criteria: Diagnostic and Statistical Manual of Mental Disorders diagnosis of OCD as assessed by the Structured-Clinical Interview for Diagnostic and Statistical Manual of Mental Disorders Axis I disorders; age between 18 and 65 years; Yale-Brown Obsessive Compulsive Scale (Y-BOCS) score >20; written informed consent; and negative serum or urinary beta-human chorionic gonadotropin test for females of childbearing potential.

Exclusion criteria involved pregnant women or women of childbearing potential who were not using medically accepted means of contraception; women who were pregnant or breastfeeding; patients who, in the investigator’s judgment, pose serious suicidal or homicidal risk; and history of a seizure disorder or other serious medical illness. Additional exclusion criteria included anticoagulant therapy or other medications that might interfere with duloxetine and patients with comorbid bipolar diagnosis, psychosis, organic mental disorders, or developmental disorders. If there was a history of substance abuse, patients must have been in remission for at least 6 months. Current treatment with behavioral therapy, specifically exposure and response prevention, for OCD excluded patients from participation. If patients were prescribed an antidepressant for major depression within the past 12 months, they were excluded. Further exclusion criteria included psychotropic medication other than a benzodiazepine or sleep aid within 2 weeks of beginning the study, more than one adequate trial (at least 10 weeks at maximally tolerated dose) with another SNRI, previously known hypersensitivity to duloxetine or any of the inactive ingredients, treatment with an monoamine oxidase inhibitor within 14 days of randomization, and patients with uncontrolled narrow-angle glaucoma.

### Schedule of Assessments and Study Instruments

Interested participants completed phone screens for study eligibility and were then evaluated at an initial visit (visit 1) for inclusion/exclusion criteria. During visit 1, the Structured-Clinical Interview for Diagnostic and Statistical Manual of Mental Disorders Axis I disorders (First et al., 1995) was administered for diagnostic evaluation. The severity of OCD symptoms was assessed using the Y-BOCS (Goodman et al., 1989b 1989c), depressive and anxiety symptoms were assessed with the Beck Depression Inventory (BDI; Beck et al., 1961) and the Beck Anxiety Inventory (BAI; Beck et al., 1988), and the Quality of Life, Enjoyment, and Satisfaction Questionnaire (Q-LES-Q; Endicott et al., 1993) was utilized to assess quality of life. Additionally, blood pressure was taken at each study visit to ensure it remained within normal ranges.

After visit 1, eligible patients began open-label treatment with duloxetine at 30mg/d. They were seen for a follow-up visit (visit 2) after 1 week. At visit 2, patients were assessed and, if they were not experiencing significant adverse events, their dose was increased from 30 to 60mg/d. Patients taking 60mg/d then returned for assessment in 4 weeks (visit 3). At this time, if they were not experiencing significant adverse events, the dose was increased to 120mg/d. If a patient was unable to tolerate 120mg/d, their dose was decreased back to 60mg/d. Remaining assessment then occurred every 4 weeks (visits 4, 5, and 6). In total, this was a 17-week study with 12 weeks at the high dose believed to be necessary for response.

At each study visit following the initial visit, patients were assessed using the Y-BOCS, BDI, BAI, and Clinical Global Improvement scale (CGI; Guy, 1976). The Q-LES-Q was administered only at the first and last study visits. The primary study outcome measures were the Y-BOCS and CGI. The Q-LES-Q was considered a secondary measure, and the BDI and BAI were considered tertiary.

### Data Analyses

Independent 2-tailed, paired *t* tests were used to assess the effects of duloxetine on all study outcome measures. Both completer and intention-to-treat (ITT) analyses were conducted. Completer analyses included subjects who completed all 6 study visits (n=12). ITT analysis included all participants who signed consent and completed visit 1 (n=20). To complete the ITT analysis, the last visit completed was carried forward and treated as the final visit. Full responders were defined as those who had a decrease in Y-BOCS score by ≥25% AND a CGI score of <3 at the final study visit. Nonresponders were those who failed to satisfy either criterion of improvement.

## Results

### Sample Characteristics

Eleven (55%) of the 20 study participants were female. The mean age at the initiation of the study was 29.90 (SD=10.66; range=18–55) years. The mean age of onset of OCD was 15.35 (SD=10.479; range=5–50) years, with a mean duration of illness of 14.80 (SD=12.88; range=1–46) years.

### ITT Analyses

Twenty participants (11 female) were included in the ITT analyses. All subjects were included in the ITT analysis, because all 20 enrolled completed their visit 1. The ITT analysis was performed using the last observation carried forward method and included all eligible participants who received their medication after visit 1. Statistically significant (*P*<.05) changes in scale scores from visit 1 to visit 6 were reported for primary and secondary outcome measures (Table 1). Mean baseline Y-BOCS score for ITT was 27.45 (SD= 4.08) and the mean final Y-BOCS score was 20.45 (SD = 7.57). CGI scores were not taken at visit 1, so values from visit 2 were compared with the final CGI. The mean CGI score at final study visit was 2.70 (SD = 0.923), which corresponds to a value between “much improved” and “minimally improved.” Mean baseline Q-LES-Q for ITT was 70.14 (SD = 14.03) and mean final score was 74.55 (SD = 12.03), giving a *t* = -2.15 and a *P* value = 0.045. Mean baseline BDI was 10.30 (SD = 6.98) and mean baseline BAI was 10.70 (SD = 11.54), with mean final BDI being 6.95 (SD = 6.10) and mean final BAI being 6.75 (SD = 7.05). BDI change was significant at *P*<.05, but BAI score was not. Of the 20 study participants, 7 (35%) were full medication responders and 9 (45%) were nonresponders.

**Table 1.** Intention-To-Treat Analyses

Rating Scale	Baseline (mean ± SD)	Final Visit (mean ± SD)	*t*	d.f.	*P* value	Cohen’s d
Y-BOCS	27.45±4.08	20.45±7.57	5.44	19	<.001	1.22
CGI	4.0±0	2.70 ± .923	6.30	19	<.001	1.41
Q-LES-Q	70.14±14.03	74.55±12.03	−2.15	19	.045	0.48
BDI	10.30±6.98	6.95±6.10	3.20	19	.005	0.72
BAI	10.70±11.54	6.75±7.05	1.67	19	.112	0.37

Intention-to-treat (ITT) analyses (n=20). All ITT analyses utilized the last observation carried forward method. BAI, Beck Anxiety Inventory; BDI, Beck Depression Inventory; CGI, Clinical Global Impressions Scale; Q-LES-Q, Quality of Life, Enjoyment, and Satisfaction Questionnaire; Y-BOCS, Yale-Brown Obsessive Compulsive Scale.

### Completer Analyses

Twelve participants were included in the completer analyses (6 female). Two participants were lost due to follow-up despite repeated attempts to contact them. One participant chose not to take the medication after completing visit 1 and signing the consent form. Five participants chose to discontinue the study because of adverse events. Statistically significant (*P*<.05) changes from visit 1 to visit 6 were reported for the primary and secondary outcome measures (Table 2). Mean baseline Y-BOCS score for completers was 28.33 (SD = 4.66) and mean final Y-BOCS score was 18.5 (SD = 7.98). At visit 6, the mean CGI score was 2.17 (SD = .72), which corresponds to a rating between “much improved” and “minimally improved.” Mean baseline Q-LES-Q for completers was 70.50 (SD = 16.53) and mean final was 77.86 (SD = 12.40), giving a *t* = -2.30 and *P* value of 0.042. Mean baseline BDI was 9.83 (SD = 8.16) and mean baseline BAI was 10.92 (SD = 13.09), with mean final BDI being 6.33 (SD = 6.77) and mean final BAI being 4.91 (SD = 5.70). BDI and BAI scores, the tertiary outcome measures, were not significant at *P*<.05. Of the 12 participants who completed the open-label duloxetine treatment, 7 (58.3%) were full medication responders and 3 (25%) were nonresponders.

**Table 2.** Completer Analyses

Rating Scale	Baseline (mean ± SD)	Final Visit (mean ± SD)	*t*	d.f.	*P* value	Cohen’s d
Y-BOCS	28.33±4.66	18.5±7.98	6.71	11	<.001	1.94
CGI	4.00±0	2.17 ± .72	8.84	11	<.001	2.55
Q-LES-Q	70.50±16.53	77.86±12.40	−2.30	11	.045	0.66
BDI	9.83±8.16	6.33±6.77	2.13	11	.056	0.62
BAI	10.92±13.09	4.91±5.70	1.80	11	.099	0.52

Completer analyses (n=12). BAI, Beck Anxiety Inventory; BDI, Beck Depression Inventory; CGI, Clinical Global Impressions Scale; Q-LES-Q, Quality of Life, Enjoyment, and Satisfaction Questionnaire; Y-BOCS, Yale-Brown Obsessive Compulsive Scale.

### Adverse Events

The most common adverse event was nausea (50% of subjects), followed by fatigue (41.2%), sexual dysfunction (23.1%), and headache (11.1%). There were no serious adverse events.

Blood pressure (measured in mmHg) was taken at each study visit. Independent, paired-samples *t* tests were run on both systolic and diastolic pressures. The baseline systolic was 122.33±13.38 for completers and 121.0±14.71 for ITT, and at the final visit the systolic pressure was 124.42±14.87 for completers and 123.1±16.04 for ITT. It was found that systolic pressure did not significantly increase for either the completer group (*P*=.561; *t*=-0.599) or the ITT group (*P*=.382; *t*=-0.895). The baseline diastolic was 71.25±9.31 for completers and 73.15±8.61 for ITT, and at the final visit the diastolic pressure was 79.83±9.61 for completers and 78.50±8.92 for ITT. Diastolic pressure did significantly increase for both the completer group (*P*=.001; *t*=-4.288) and the ITT group (*P*=.008; *t*=-2.944).

## Discussion

The results of this study support the efficacy of duloxetine in reducing severity of OCD symptoms, as 58.3% of the subjects who completed the study were full medication responders. Statistically significant improvement was reported for the primary and secondary outcome measures for both the completer and ITT analyses. However, in the ITT group, the observed 7-point drop in Y-BOCS score is moderate. It should be noted that adverse events were relatively common and that 40% of the subjects enrolled dropped out. However, duloxetine was relatively well tolerated by our completer study sample, with only 2 participants requiring a reduction in dosage due to adverse events.

Although there are few data on SNRIs for the treatment of OCD, these previous data have suggested that SNRIs will prove useful for relief of OCD symptoms. The additional noradrenergic effects of the SNRIs could provide an additive advantage in treatment. Specifically, affecting multiple neurotransmitter systems could have additive value for the treatment of comorbid disorders. However, based on the frequency of adverse events observed in this clinical trial, duloxetine may be less tolerable than other SRIs and is certainly less tolerable than behavioral therapy. Duloxetine is indicated for the treatment of major depressive disorder, which is commonly comorbid with OCD. Furthermore, duloxetine is helpful for managing symptoms of chronic pain. Somatic-obsessions, sometimes associated with real or imagined pain, are common in OCD. Also, symptoms of pain (eg, headaches and muscle tension) are common physical manifestations of anxiety. Duloxetine is FDA approved for management of chronic lower back pain, osteoarthritis pain, diabetic peripheral neuropathic pain, and musculoskeletal pain. Given these indications, duloxetine is unique among SNRIs and therefore has promising implications as a novel pharmacological treatment of OCD.

There are several weaknesses in this trial limiting the interpretation of the data. The study was a small sample, with a recruitment goal of only 20 individuals, and an anticipated drop-out rate of 40%. The 20 participants were consecutively recruited from 30 completed phone screens. Of the 30 phone screens, 13 were male and 17 were female. Two of the 20 participants had comorbid Generalized Anxiety Disorder diagnoses. Excluding those 2 participants from *t* tests for Y-BOCS, BDI, BAI, CGI, and Q-LES-Q results did not change for either ITT or completer analyses. Also, the study was open-label, such that both investigators and participants were aware of the dosage they were receiving. This could have led to a placebo effect, increasing the response rate as subjects received increased doses of duloxetine. Further research will be required to establish the optimal dosage of duloxetine for individual treatment.

Despite the limitations of the trial, this study is the first open-label investigation of duloxetine for the treatment of OCD. Double-blind, placebo-controlled studies with larger sample sizes are needed to further evaluate the efficacy of duloxetine for OCD. However, this investigation offers promising results for the use of an SNRI as treatment for OCD.

## Statement of Interest

Dr. Dougherty has served as a consultant to Medtronic, received honoraria from Medtronic, received travel support from Roche, and received grant/research support from Medtronic, Cyberonics, Eli Lilly, and Roche. Dr. Keuthen has received grant/research support from the Trichotillomania Learning Center, is on the advisory board of the International Obsessive Compulsive Disorder Foundation and the Trichotillomania Learning Center, is a stock shareholder of Pfizer Inc., Merck & Co. Inc., GlaxoSmithKline, and Johnson & Johnson, and has received royalties from New Harbinger.

## References

[CIT0001] Ananth J Burgoyne K Smith M Swartz R (1995) Venlafaxine for the treatment of obsessive-compulsive disorder [letter]. Am J Psychiatry 152:1832. 8526258 10.1176/ajp.152.12.1832a

[CIT0002] Beck AT Ward CH Mendelson M Mock J Erbaugh J (1961) An inventory for measuring depression. Arch Gen Psychiatry 4:561–571. 13688369 10.1001/archpsyc.1961.01710120031004

[CIT0003] Beck AT Epstein N Brown G Steer RA (1988) An inventory for measuring clinical anxiety: the Beck Anxiety Inventory. J Consult Clin Psychol 56:893–897. 3204199 10.1037//0022-006x.56.6.893

[CIT0004] Denys D van der Wee N van Megen HJ Westenberg HG (2003) A double blind comparison of venlafaxine and paroxetine in obsessive-compulsive disorder. J Clin Psychopharmacol 23:568–575. 14624187 10.1097/01.jcp.0000095342.32154.54

[CIT0005] Dougherty DD Rauch SL Jenike MA (2004) Pharmacotherapy for obsessive-compulsive disorder. J Clin Psychol 60:1195–1202. 15389617 10.1002/jclp.20083

[CIT0006] Endicott J Nee I Harrison W Blumenthal R (1993) Quality of Life, Enjoyment, and Satisfaction Questionnaire: a new measure. Psychopharmacol Bull 29:321–326. 8290681

[CIT0007] First MB Spitzer RL Gibbon M Williams JBW (1995) Structured Clinical Interview for the DSM-IV-TR Axis I Disorders-Patient Edition (SCID-I/P, 11/2002 revision). New York: Biometric Research, New York State Psychiatric Institute.

[CIT0008] Goodman WK Price LH Rasmussen SA Delgado PL Heninger GR Charney DS (1989a) Efficacy of fluvoxamine in obsessive-compulsive disorder: a double-blind comparison with placebo. Arch Gen Psychiatry 46:36–44. 2491940 10.1001/archpsyc.1989.01810010038006

[CIT0009] Goodman WK Price LH Rasmussen SA Mazure C Fleischman RL Hill CL Heninger GR Charney DS (1989b) The Yale-Brown Obsessive Compulsive Scale. I. Development, use, and reliability. Arch Gen Psychiatry 46:1006–1011. 2684084 10.1001/archpsyc.1989.01810110048007

[CIT0010] Goodman WK Price LH Rasmussen SA Mazure C Delgado P Heninger GR Charney DS (1989c) The Yale-Brown Obsessive Compulsive Scale. II. Validity. Arch Gen Psychiatry 46:1011–1016. 10.1001/archpsyc.1989.01810110054008 2510699

[CIT0011] Goodman WK Kozak MJ Liebowitz M White KL (1996) Treatment of obsessive-compulsive disorder with fluvoxamine: a multicentre, double-blind, placebo-controlled trial. Int Clin Psychopharmacol 11:21–29. 8732310

[CIT0012] Grossman R Hollander E (1996) Treatment of obsessive-compulsive disorder with venlafaxine. Am J Psychiatry 153:576–577. 8599411 10.1176/ajp.153.4.576b

[CIT0013] Guy W (1976) ECDEU Assessment Manual for Psychopharmacology. US Dept of Health, Education, and Welfare publication (ADM) 76–338, pp 218–222. Rockville, MD: National Institute of Mental Health.

[CIT0014] Jenike MA Baer L Summergrad P Minichiello WE Holland A Seymour R (1990) Sertraline in obsessive-compulsive disorder: a double-blind comparison with placebo. Am J Psychiatry 147:923,928. 2192564 10.1176/ajp.147.7.923

[CIT0015] Karabanow O (1977) Double-blind controlled study in phobias and obsessions. J Int Med Res 5(Suppl 5):42–48. 340306

[CIT0016] Kronig MH Apter J Asnis G Bystritsky A Curtis G Ferguson J Landbloom R Munjack D Riesenberg R Robinson D Roy-Byrne P Phillips K Du Pont IJ (1999) Placebo-controlled, multicenter study of sertraline treatment for obsessive-compulsive disorder. J Clin Psychopharmacol 19:172–176. 10211919 10.1097/00004714-199904000-00013

[CIT0017] Montgomery SA (1980) Clomipramine in obsessional neurosis: a placebo-controlled trial. Pharmaceutical Med 1:189–192.

[CIT0018] Perse TL Greist JH Jefferson JW Rosenfeld R Dar R (1987) Fluvoxamine treatment of obsessive-compulsive disorder. Am J Psychiatry 144:1543–1548. 3120604 10.1176/ajp.144.12.1543

[CIT0019] Rauch SL O’Sullivan RL Jenike MA (1996) Open treatment of obsessive-compulsive disorder with venlafaxine: a series of ten cases. J Clin Psychopharmacol 16:81–84. 8834427 10.1097/00004714-199602000-00017

[CIT0020] Zajecka JM Fawcett J Guy C (1990) Coexisting major depression and obsessive-compulsive disorder treated with venlafaxine. J Clin Psychopharmacol 10:152–153. 2341595 10.1097/00004714-199004000-00033

[CIT0021] Zhao JP (1991) A controlled study of clomipramine and amitriptyline for treating obsessive-compulsive disorder. Chung Hua Shen Ching Shen Ko Tsa Chih 24:68–70. 1860383

